# Preparation of Al_3_Ti-Al_2_O_3_/Al Inoculant and Its Inoculation Effect on Al-Cu-Mn Alloy

**DOI:** 10.3390/ma16155264

**Published:** 2023-07-27

**Authors:** Jinhua Ding, Chao Wang, Cheng Lu, Guangming Zhu, Nana Guo, Xujie Gao, Xin Wang, Chunxiang Cui

**Affiliations:** 1 School of Mechanical Engineering, Shandong University of Technology, Zibo 255000, China; 2 School of Physics and Optoelectronic Engineering, Shandong University of Technology, Zibo 255000, China; 3 Hebei Key Laboratory of New Functional Materials, School of Material Science and Engineering, Hebei University of Technology, Tianjin 300401, China

**Keywords:** inoculant, Al_2_O_3_ whiskers, grain refinement, microstructure, property

## Abstract

The grain size plays a pivotal role in determining the properties of the alloy. The grain size can be significantly decreased by adding inoculants. Aiming to address the shortcomings of existing inoculants, the Al_3_Ti-Al_2_O_3_/Al inoculant was successfully prepared using Al-Ti master alloy and Al_2_O_3_ whiskers as raw materials. With the aid of ultrasonic energy, the Al_2_O_3_ whiskers were uniformly dispersed within the inoculants. Under the combined action of ultrasonic and titanium, the Al_2_O_3_ whiskers were broken into small particles at high temperature. To enhance the morphology of Al_3_Ti and achieve even particle dispersion throughout the matrix, vacuum rapid quenching treatment was applied to the inoculant. The SEM test results indicated a significant reduction in particle size after vacuum rapid quenching. The Al_3_Ti-Al_2_O_3_/Al inoculants exhibited excellent grain refinement effects on the weldable Al-Cu-Mn alloy. Crystallographic calculations and HRTEM analysis revealed that Al_2_O_3_ and Al have orientation relationships, indicating their potential as effective heterogeneous nucleation sites. The mechanical properties of the Al-Cu-Mn alloy were obviously improved after the Al_3_Ti-Al_2_O_3_/Al inoculant was added.

## 1. Introduction

The refinement of grain is widely acknowledged as a straightforward and efficient approach to enhance the microstructure and properties of aluminum alloys [1,2,3,4,5]. Grain refinement can be achieved through adding inoculants, increasing melt cooling rate, stirring, ultrasonic treatment, plastic deformation and heat treatment [6,7,8,9,10]. Among these refining methods, adding inoculants to aluminum alloy melt is the most simple and effective method. The inoculants of aluminum alloy mainly include some alloying elements and ceramic particles [11,12,13,14]. Alloying elements usually play a role in heterogeneous nucleation of Al matrix by forming intermetallic compounds with Al. The main requirements for ceramic inoculants are high melting points, excellent physical and chemical stability, and low lattice misfit with the substrates [15,16,17,18,19,20].

For most aluminum alloys, the Al-5Ti-B refiner can play a role in grain refinement. However, in the Al-5Ti-B ingot, TiB_2_ particles tend to aggregate and form clusters, and the Al_3_Ti phase is usually needle-like, which are detrimental to the refinement effect. Moreover, the presence of elements such as Zr, Si, Cr, and V in aluminum alloy can lead to a “poisoning” effect on Al-5Ti-B [21,22,23,24]. For instance, even 0.2 wt.% of Zr can rapidly diminish the refining capability of Al-5Ti-B. The reason is the formation of (Ti_1−x_Zr_x_)Al_3_ and (Ti_1−x_Zr_x_)B_2_, which leads to the weakening of heterogeneous nucleation ability of TiB_2_ and Al_3_Ti particles. Additionally, the formation of (Ti_1−x_Zr_x_)Al_3_ weakens the constraining effect of free Ti on grain growth, while Zr disrupts the two-dimensional monatomic layer of Al_3_Ti on the surface of TiB_2_, resulting in a loss of grain refinement effectiveness [25,26,27].

Then, Al-Ti-B-RE [28,29] was developed to further improve the refining effect of Al-Ti-B refiner. The addition of rare earth can not only change the morphology of Al_3_Ti particles but also improve the dispersibility of TiB_2_ and Al_3_Ti particles and increase the degree of supercooling of aluminum melt. Compared with the traditional Al-Ti-B refiner, Al-Ti-B-RE refiner has a better refining effect, but its optimization effect is limited, the process is complicated and the cost is high.

A1-Ti-C intermediate alloy is also an aluminum alloy refiner widely used in industrial production. In crystallography, there exists a specific orientation relationship between TiC particles and the α-Al matrix, and so, it has heterogeneous nucleation potential for α-Al. Li et al. [30] found that the dispersion of TiC in aluminum matrix is obviously better than that of TiB_2_ particles, and the particle size of TiC is much smaller than that of TiB_2_. Therefore, for two kinds of particles with the same mass fraction, the number of TiC particles is more than that of TiB_2_, so that more nucleation sites can be provided for α-Al, thus achieving a better refining effect. However, the major disadvantage of A1-Ti-C is that TiC and Al will react to form Al_4_C_3_ brittle phase at a certain temperature: 13Al (l) +3TiC (s) = 3TiAl_3_ (s) + Al_4_C_3_ (s) [31], resulting in poor stability of TiC and fading behaviors of the refinement effect. In addition, the preparation of Al-Ti-C refiner is usually based on graphite powder, carbon tetrachloride and other carbon sources, which are added to the Al-Ti intermediate alloy melt at high temperature and then reacted with each other. However, due to the poor wettability of carbon in aluminum, the low utilization rate of C and the high reaction temperature, the required preparation equipment is complicated and the cost is high. Therefore, Al-Ti-C refiner cannot be used on a large scale in industrial production [32].

In order to improve the refining effect of Al-Ti-C refiner, Zhao, Ding and Xu et al. [33,34] prepared Al-Ti-C-RE refiner. After the addition of rare earth elements, the wettability of C and Al melt is improved, which promotes the formation of TiC particles. In addition, rare earth can improve the morphology of Al_3_Ti particles, reduce the size of Al_3_Ti particles, improve the dispersion of TiC and Al_3_Ti particles and improve the refining effect and property of anti-degeneration of refiners. However, the development of Al-Ti-C-RE refiner is the same as that of Al-Ti-B-RE refiner, with limited optimization effect, cumbersome process and high cost [35,36,37]. Therefore, it is necessary to develop new inoculants to overcome the shortcomings of existing inoculants.

α-Al_2_O_3_ belongs to the trigonal system and is composed of hexagonal tightly packed crystals. The lattice constant of α-Al_2_O_3_ is a = b = 0.4759 nm, c = 1.3 nm. According to the edge-to-edge model proposed by Zhang et al. [17], the orientation relationships between α-Al and α-Al_2_O_3_ are as follows: (200)_Al_||(006)_Al_2_O_3__, [011]_Al_||[$\bar{1}$20]; (200)_Al_||(006)_Al_2_O_3__, [011]_Al_||[$\bar{1}$10]_Al_2_O_3__; (111)_Al_||(011)_Al_2_O_3__, [01$\bar{1}$]_Al_||[11$\bar{1}$]_Al_2_O_3__. In addition, α-Al_2_O_3_ has the advantages of high strength, high melting point, high elastic modulus and good stability [38,39,40,41,42]; as a result, it can serve as heterogeneous substrates for α-Al and effectively refine aluminum alloys. In this paper, we desired to replace TiB_2_ with Al_2_O_3_ to overcome the shortcomings of the existing inoculants.

The wettability between α-Al_2_O_3_ particles and liquid aluminum is poor, and so, it is easy to aggregate into clusters on the surface of liquid aluminum. In this paper, the Al_2_O_3_ whiskers instead of Al_2_O_3_ particles were added to the liquid Al-Ti master alloy at high temperature, and ultrasonic treatment was used to improve the dispersion and wettability.

## 2. Materials and Methods

The primary equipment utilized in the preparation of Al-Cu-Mn alloy was predominantly the MZG series high-frequency induction melting furnace. First, commercial pure aluminum (99 wt.%), commercial pure copper (99 wt.%), commercial pure zinc (99 wt.%), Al-Mn, Al-Ti, Al-Zr and Al-V were proportionally weighed using an electronic scale. The weighed pure aluminum and pure copper were placed in the graphite crucible and heated in the MZG series high-frequency induction melting furnace. After these two metals were melted, the weighed commercial pure zinc (99 wt.%), Al-Mn, Al-Ti, Al-Zr and Al-V were quickly put in and fully stirred with graphite rods. After all the raw materials were melted and fully stirred, the dross on the surface of the molten liquid was removed with a scraping spoon and was then poured into the preheated steel mold to produce alloy sample rods with Φ20 mm × 100 mm.

The raw materials for preparation of Al_3_Ti-Al_2_O_3_/Al inoculant were Al-Ti master alloy and alumina whiskers. The alumina whiskers were provided by the Shenzhen Research Institute of Tsinghua University (Shenzhen, China). The Al-Ti master alloy was melted in a high frequency induction furnace, and then the alumina whiskers were added to the melt in a certain proportion. To make the whisker evenly dispersed in the melt, ultrasonic vibration treatment was applied to the melt, and the metal liquid was then cast into the steel mold. To achieve a uniform dispersion of the inoculant in the Al-Cu-Mn matrix alloy, the inoculant ingots were remelted and transformed into ribbons through vacuum rapid quenching, that is, by pouring liquid metal on a spinning cold copper wheel.

The Al-Cu-Mn alloy was melted at 800 °C in the crucible resistance furnace. After the Al-Cu-Mn alloy was melted, the inoculant ribbons were added to the melt and thoroughly stirred with a graphite rod and were then cast into the steel mold of Φ20 mm × 100 mm. The Al-Cu-Mn alloys underwent T6 heat treatment, which involved solution treatment at 520 °C for 24 h followed by water quenching and artificial aging treatment at 165 °C for 14 h.

The phase composition of the inoculants was determined via X-ray diffraction (XRD). The metallographic microscope was utilized to observe the changes in grain morphology and size. The microstructures and element composition of the alloy and inoculant were analyzed by the scanning electron microscopy (SEM) equipped with energy dispersive X-ray spectroscopy (EDS). To enable observation of the sample under both optical and scanning electron microscopes, it underwent a series of preparation steps including sandpaper polishing with varying roughness, mechanical polishing using a specialized machine and, finally, corrosion treatment utilizing Keller reagent. The heterogeneous nucleation particles in alloy were characterized using JEM-2100 F (JEOL, Tokyo, Japan) transmission electron microscope (TEM). The specimens for TEM observation were prepared by ion milling. The mechanical properties of the alloy was measured using a universal testing machine.

## 3. Results and Discussion

**Figure 1:**
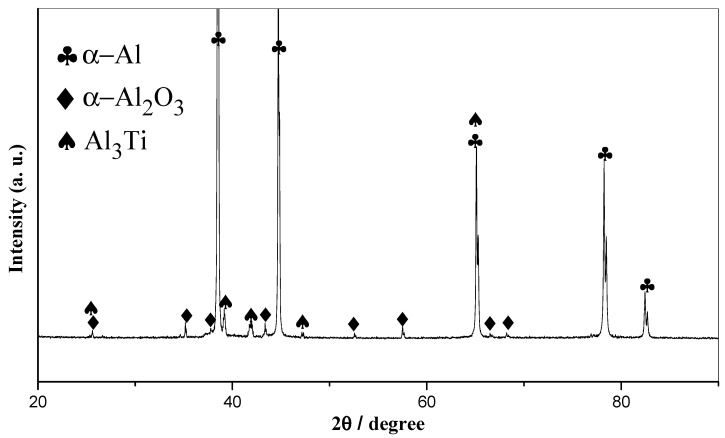
XRD pattern of Al_3_Ti-Al_2_O_3_/Al inoculant.

The XRD pattern of Al_3_Ti-Al_2_O_3_/Al inoculant is presented in Figure 1, revealing that the phase composition of the inoculant primarily comprised α-Al, α-Al_2_O_3_ and Al_3_Ti.

**Figure 2:**
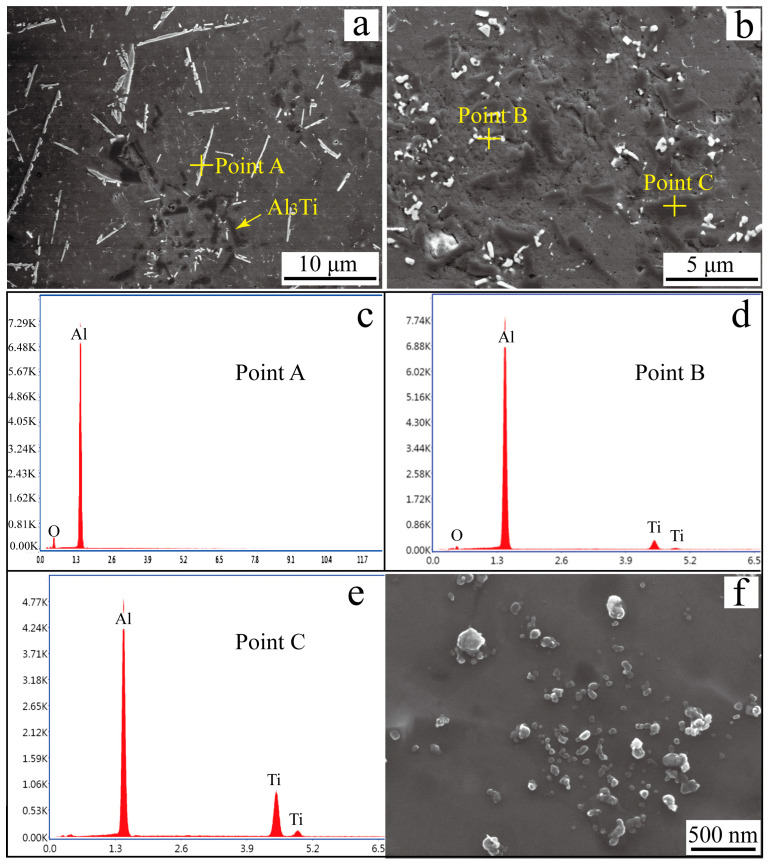
(**a**,**b**) SEM images of Al_3_Ti-Al_2_O_3_/Al inoculants, (**c**) EDS pattern of Point A, (**d**) EDS pattern of Point B, (**e**) EDS pattern of Point C, (**f**) SEM image of Al_3_Ti-Al_2_O_3_/Al inoculant after vacuum rapid quenching.

The SEM images of Al_3_Ti-Al_2_O_3_/Al inoculants and the EDS patterns of the second phases are presented in Figure 2. The matrix contained distributed rod-like and granular second phases, as illustrated in Figure 2a. In Figure 2b, it can be seen that the granular second phases were nanometer and submicron in size. The rod-like second phases were alumina whiskers, and it can be found that there were craters on the surface of the alumina whiskers and the whisker length was obviously reduced. At high temperature (1600~1700 °C), Al_2_O_3_ will decompose and release O atoms, the O atoms can easily combine with the Ti atoms in the melt to form TiO_2_ or Al_2_TiO_5_ [43,44,45]. However, no diffraction peaks corresponding to other phases were observed in the XRD pattern shown in Figure 1, probably because too few reaction products were generated to be detected. Because Al_2_O_3_ can react with titanium at high temperatures, titanium in the Al-Ti master alloy will corrode the whisker and break it. The EDS pattern of point B indicates that the particles in Figure 2b were the chips dropped from the alumina whiskers after being fragmented. The gray phases in Figure 2a,b are Al_3_Ti, and it can be seen that before the vacuum rapid quenching treatment, its morphology was short rod. The refining effect of large particles was less than ideal when compared to that of fine particles. Therefore, to achieve optimal refining results, the inoculant ingot was rapidly quenched under vacuum conditions. The SEM image of the Al_3_Ti-Al_2_O_3_/Al ribbon in Figure 2f reveals a disappearance of large rod-like Al_3_Ti particles and an emergence of numerous gray nanoparticles. In the process of remelting, the Al_3_Ti particles were dissolved in liquid aluminum. In the subsequent solidification stage, due to the rapid cooling rate, these particles did not have sufficient time to grow and, thus, resulted in the formation of numerous nanoparticles.

**Figure 3:**
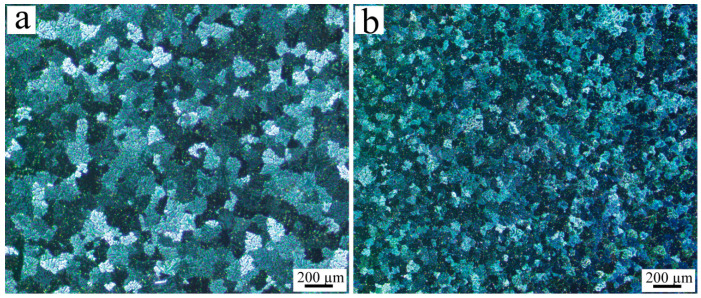
Metallographic image of (**a**) Al-Cu-Mn alloy, (**b**) Al-Cu-Mn alloy inoculated by Al_3_Ti-Al_2_O_3_/Al.

To evaluate the refining effect of Al_3_Ti-Al_2_O_3_/Al inoculant on Al-Cu-Mn alloy, 1 wt.% of the inoculant was introduced into the alloy. The metallographic images of the Al-Cu-Mn alloy, both pre- and post-inoculation, are depicted in Figure 3. The addition of the inoculant resulted in a significant reduction in grain size for the Al-Cu-Mn alloy. The main reasons for the decrease in the grain size after adding Al_3_Ti-Al_2_O_3_/Al inoculant were as follows: first, Al_3_Ti can serve as a heterogeneous nucleation site for α-Al; second, Ti can limit the growth of aluminum grains; third, the calculation results show that Al_2_O_3_ and Al also had crystal orientation relationships and, therefore, it can serve as a site for heterogeneous nucleation of α-Al during the solidification process of the alloy.

**Figure 4:**
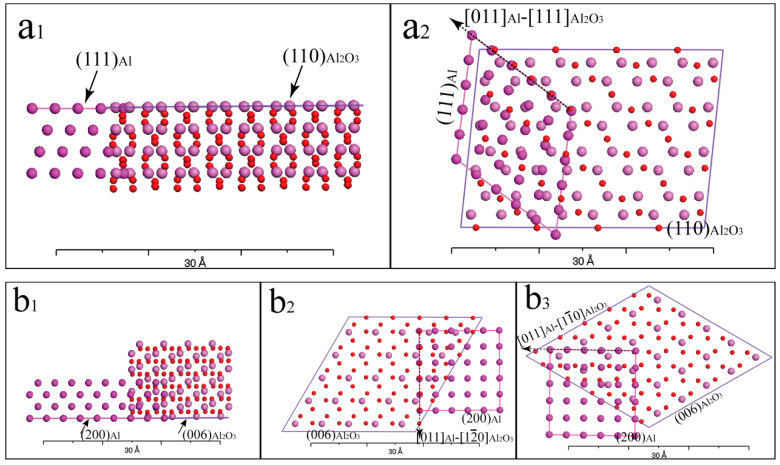
Schematic diagram of the lattice matching between Al and Al_2_O_3_. (**a_1_**) (111)_Al_//(110)_Al_2_O_3__; (**a_2_**) [011]_Al_//[111]_Al_2_O_3__; (**b_1_**) (200)_Al_//(006)_Al_2_O_3__; (**b_2_**) [011]_Al_//[1$\bar{2}$0]_Al_2_O_3__; (**b_3_**) [011]_Al_//[1$\bar{1}$0]_Al_2_O_3__.

The (111), (200) and (220) planes of Al corresponded to the first, second and third close-packed planes, respectively, with interplanar spacings of 0.234 nm, 0.202 nm and 0.143 nm. The close-packed crystallographic planes of Al_2_O_3_ were (012), (104), (110) and (006) with corresponding interplanar spacings of 0.348 nm, 0.255 nm, 0.238 nm and 0.217 nm, respectively. By calculating, Al and Al_2_O_3_ can form the following orientation relationships: (111)_Al_//(110)_Al_2_O_3__, [011]_Al_//[111]_Al_2_O_3__; (200)_Al_//(006)_Al_2_O_3__, [011]_Al_//[1$\bar{2}$0]_Al_2_O_3__; (200)_Al_//(006)_Al_2_O_3__, [011]_Al_//[1$\bar{1}$0]_Al_2_O_3__. The Materials Studio (MS 7.0) software was utilized to generate a schematic diagram illustrating the lattice matching between Al_2_O_3_ and Al, as depicted in Figure 4. In Figure 4(a_1_,a_2_), it can be seen the crystal plane spacing of (111)_Al_ was close to that of (110)_Al_2_O_3__, the atomic spacing of [011]_Al_ was close to that of [111]_Al_2_O_3__; therefore, they can form a good interface combination in theory. In Figure 4(b_1_–b_3_), it can be seen that when the (200)_Al_ was combined with the (006)_Al_2_O_3__, the plane spacing between them and the atomic spacing of the corresponding crystal orientation were very close, and so, (200)_Al_ and (006)_Al_2_O_3__ also can form a good interface combination in theory.

**Figure 5:**
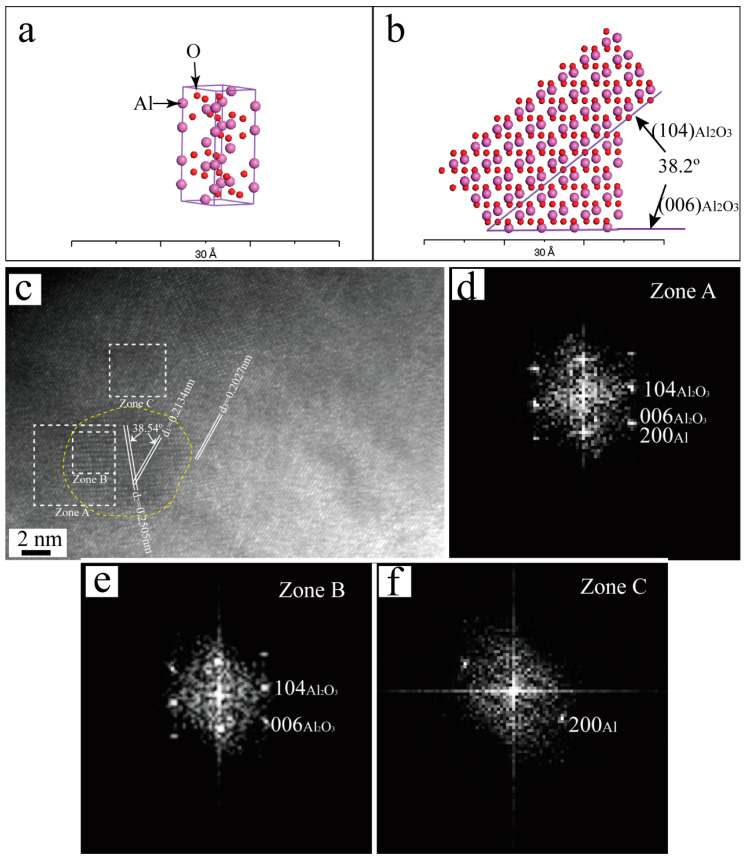
(**a**,**b**) Al_2_O_3_ lattice and crystal plane simulation diagrams, (**c**) HRTEM image of Al_2_O_3_ particle, (**d**–**f**) fast Fourier transform (FFT) patterns of zone A, zone B and Zone C.

The HRTEM image of the Al-Cu-Mn alloy inoculated with the Al_3_Ti-Al_2_O_3_/Al inoculant is presented in Figure 5c, revealing a slight variation in atomic arrangement within the circular box region compared to its surrounding area. The measurement results show that the interplanar spacing d_1_ was 0.2134 nm and d_2_ was 0.2505 nm, and the angle between the two crystallographic planes was 38.54°. Figure 5a is the simulation of Al_2_O_3_ lattice built by MS 7.0 software. Figure 5b is the simulation of (104) and (006) planes cut from Al_2_O_3_ lattice, the plane spacing of (104) and (006) was 0.255 nm and 0.217 nm, respectively, and the angle between them was 38.2°. It indicates that the phase of the round frame region corresponded to the Al_2_O_3_, and so, the round frame area can be identified as Al_2_O_3_. Figure 5d–f depict the fast Fourier transform (FFT) patterns of zones A, B and C. As evidenced by the HRTEM image and FFT pattern, the (006)_Al_2_O_3__ plane was parallel to (200)_Al_ with closely matched plane spacing. The analysis results indicate that a perfect interface can be formed between Al_2_O_3_ and Al, thus enabling Al_2_O_3_ to serve as the heterogeneous nucleation site for Al. These test findings are consistent with the aforementioned theoretical analysis.

**Figure 6:**
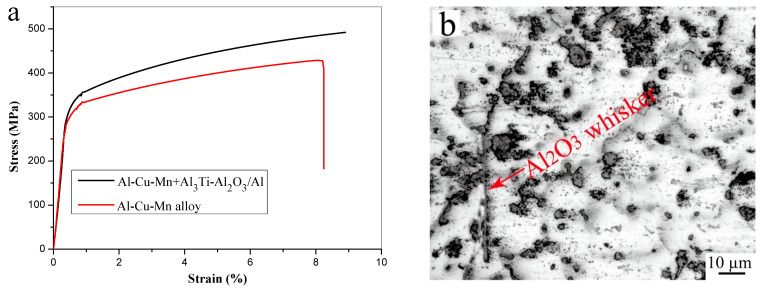
(**a**) Stress–strain curves of Al-Cu-Mn alloy and Al-Cu-Mn alloy + Al_3_Ti-Al_2_O_3_/Al inoculant, (**b**) metallographic image of Al-Cu-Mn alloy inoculated by Al_3_Ti-Al_2_O_3_/Al.

Figure 6a illustrates the stress–strain behavior of Al-Cu-Mn alloy and Al-Cu-Mn alloy treated with the Al_3_Ti-Al_2_O_3_/Al inoculant. The enhancement of the ultimate tensile strength and a slight increase in elongation were evidently observed in the alloy. The Al-Cu-Mn alloy exhibited an initial tensile strength of 428 MPa and an elongation of 8.2%. Upon inoculation with the Al_3_Ti-Al_2_O_3_/Al inoculant, the ultimate tensile strength was enhanced to 492 MPa, while the elongation increased to 8.8%. According to Figure 3, the addition of Al_3_Ti-Al_2_O_3_/Al inoculants resulted in a significant reduction in the average grain size of Al-Cu-Mn alloy, leading to an increase in grain boundaries. During plastic deformation of the alloy, the presence of grain boundaries impeded dislocation movement, thereby increasing the tensile strength of the alloy. In addition, the Al_3_Ti and Al_2_O_3_ particles can also act as the obstacles to the dislocation motion, thus leading to higher tensile strength. The alloy could maintain good plasticity while the tensile strength was increased, on the one hand because the grain refinement made the applied stress more evenly dispersed in each grain and, thus, not making it easy to form stress concentration. On the other hand, unreacted Al_2_O_3_ whiskers can serve as a bridging agent in the matrix (as illustrated in Figure 6b), thereby contributing positively to the plasticity of the alloy.

## 4. Conclusions

In this study, the Al_3_Ti-Al_2_O_3_/Al inoculant was prepared with Al-Ti master alloy and Al_2_O_3_ whiskers as raw materials. The Al_3_Ti-Al_2_O_3_/Al inoculants were used to inoculate the Al-Cu-Mn alloy and showed good grain refining effect. The key findings can be succinctly summarized as follows:

(1) Under the combined action of high temperature, ultrasonic and Ti element, parts of the Al_2_O_3_ whiskers were fragmented into granular form. After the vacuum-quenching treatment, the large sizes Al_3_Ti were replaced by small size particles.
(2) There exist orientation relationships between α-Al_2_O_3_ particles and the aluminum matrix, whereby the former can serve as heterogeneous nucleation substrates for the latter. The Al_3_Ti-Al_2_O_3_/Al inoculants exhibited a significant grain refining effect on the Al-Cu-Mn alloy.
(3) The tensile strength of the Al-Cu-Mn alloy was improved from 428 MPa to 492 MPa and the elongation was enhanced from 8.2% to 8.8% after being inoculated by the Al_3_Ti-Al_2_O_3_/Al inoculants.

## Data Availability

Any further detailed data may be obtained from the authors upon a reasonable request.
